# The Investigation of Structure-Activity Relationships of Tacrine Analogues: Electronic-Topological Method

**DOI:** 10.2174/1874104500802010075

**Published:** 2008-08-06

**Authors:** Murat Saracoglu, Fatma Kandemirli

**Affiliations:** 1 Faculty of Education, Erciyes University, 38039, Kayseri, Turkey; 2 Department of Chemistry, Kocaeli University, 41000, Izmit, Turkey

**Keywords:** Tacrine analogues, AChE, structure-activity relationships, electronic-topological method.

## Abstract

In this study we investigated the structure-activity relationships by using the Electron- Topological Method (ETM) for a class of AChE inhibitors related to tacrine (9-amino-1,2,3,4-tetrahydroacridine) and 11 *H*-Indeno-[1,2-b]-quinolin-10-ylamine that tetracyclic tacrine analogues, a drug currently in use for the treatment of the AD. Molecular fragments being specific for active and inactive compounds were revealed by using ETM. The result of testing showed the high ability of ETM in predicting the activity and inactivity in investigated series.

## INTRODUCTION

Alzheimer’s disease (AD) is a progressive neurodegenerative illness that affects up to 5% of people over 65 years, rising to 20% of those over 80 years [1]. The study of new agents useful to treat AD is one of the most active research fields in both pharmaceutical industry and academia. AD is a progressive neurodegenerative syndrome associated with aging leading to the most common form of senile dementia. The disease is characterized by the presence of some neuropathological markers detected in the brain of AD patients, which are the *β*-amyloid (*β*A) plaques and the neurofibrillary tangles. A pathogenic role is ascribed to these lesions, and many research programs focused on drugs able to modify the course of the disease are targeting both their formation and neuro-toxicity [2].

One of the few undisputed evidences in the neuropathology of the Alzheimer's disease is the loss of cholinergic neurons occurring in different areas of the central nervous system (CNS), mainly the cerebral cortex and hippocampus. This loss of cholinergic innervations is the ultimate cause of the cognitive and behavioral abnormalities that characterize AD, and it is not a surprise that the early pharmacological approaches to the treatment of the AD patients were aimed at increasing the availability of the cholinergic neuro-transmitter acetylcholine (ACh) [3]. On this basis, the cholinergic hypothesis became the leading strategy for the development of AD drugs [4, 5]. Tacrine was the first acetylcholinesterase (AChE) inhibitor launched in 1993 as the first drug for the symptomatic treatment of AD drug [6].

**Scheme 1:**
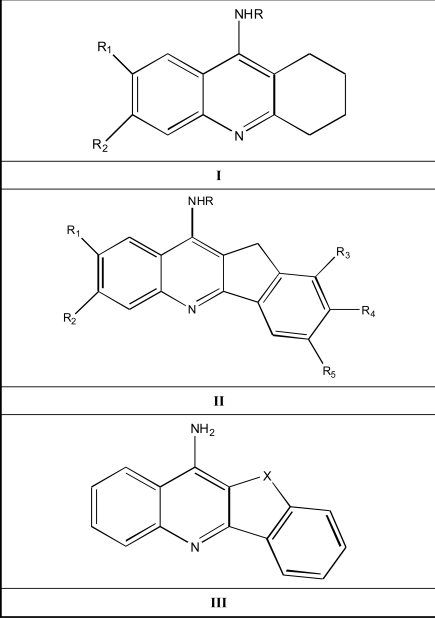
Common molecular skeletons of tacrine and 11Hindeno-[1,2-b]-quinolin-10-ylamine tetracyclic tacrine analogues.

In this study we investigated the structure-activity relationships by using the Electron- Topological Method [7-17] for a class of AChE inhibitors related to tacrine (9-amino-1, 2, 3, 4-tetrahydroacridine) analogues [6] and 11 *H*-Indeno-[1,2-b]-quinolin-10-ylamine analogues that tetracyclic tacrine analogues [18, 19], a drug currently in use for the treatment of the AD. The series under investigation were given in Table **1** and their common skeletons were shown in the Scheme **1**.

All conformational and quantum-chemical data were obtained by means of the MMP2 method of the molecular mechanics and a semi-empirical quantum-chemistry method known as AM1. Activity features’ selection has been carried out by means of the ETM-software. To have more stable activity features, every active compound was used as a template for comparison with the rest of compounds. As a result of this comparison, activity features (pharmacophores) Ph1, Ph2 and Ph3 were revealed. To decide which of pharmacophores is better, each inactive compound was used as a template for comparison with the rest of compounds. So, inactivity features (anti-pharmacophores) APh1, APh2 and APh3, were revealed also to complete the system for the AChE inhibitory activity prediction.

## MATERIALS AND METHODS

There are many methods for studying Structure-Activity Relationships (SAR), and all of them have some disadvantages. The purpose of the ETM [7-17] is to overcome the molecular descriptions of the previously developed SAR methods.

**Fig. (1):**
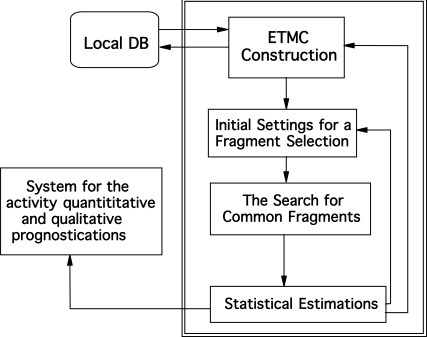
Common scheme of the ETM.

To apply ETM, activity data (qualified at least as being active or inactive) and structures of compounds are taken from outer databases or literature. Ideally, half of the molecules should be active [20]. The following steps were applied for the ETM procedure (see Fig. **1**) [8, 10, 11]:

1. Molecular mechanics conformational analyses on each of molecules were carried out [21].
2. The electronic structures of each of these conformations were calculated with semi-empirical method known as AM1.
3. Corresponding electronic and geometric parameters were arranged in a matrix *n x n *(*n *is the number of) called ETMC.
4. Series studied were divided as active, inactive and low active molecules.
5. Template molecules (the most active one) that are to be compared with the rest of molecules in the series are selected for active and inactive groups.
6. Structural fragments common to all active molecules are searched for through the comparison of all ETMCs with the template ETMC selected. Taken into account some limiting values were ± 0.05 for δ_1_ and ± 0.15 for δ_ 2_. A probabilistic criterion, P which was calculated by the following formula, is commonly used in structural methods for estimate activity fragment in a series under study.

*P*= (*n*_A_+1)/ (*n*_A_+*n*_IA_+2)

where *n*_A_, *n*_IA_ are numbers of active/inactive molecules, respectively, which contain the fragments

## RESULTS AND DISCUSSION

In this study, we used optimized geometry data and electronic characteristics to form ETMCs for all compounds in a series of to tacrine (9-amino-1, 2, 3, 4-tetrahydroacridine) analogues [6] and 11 *H*-Indeno-[1,2-b]-quinolin-10-ylamine analogues [18, 19] including 44 molecules. Effective charges on atoms are shown on diagonal elements, bond characteristics and optimized distances are represented on non diagonal elements.

According to the activity level, molecules under study (44 in all) [6, 18, 19] were divided into 3 groups:

1. Active compounds (20 mol. with IC_50_ ≤ 1.3);
2. Low active compounds (4 mol. with 1.3 > IC_50_ < 3.7);
3. Inactive compounds (20 mol. with IC_50_ ≥ 3.7) compounds.

The parameters responsible for the activity form a matrix called electron topological sub matrix of activity (ETSA), calculated from an ETMC that represents one of the most active compounds (“a template” for comparison).

### Determination of Pharmacophores Features

For each template compound, its ETMC was compared with the ETMCs of the rest of the compounds in both series. The comparison resulted in a few common structural fragments for the two cases. The fragments were found as sub-matrices of the corresponding template ETMCs (i.e. electron-topological sub-matrices of contiguity, or ETSCs, for short).

**Fig. (2):**
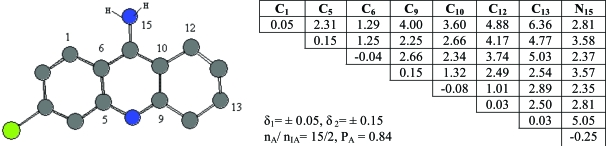
ETSC and corresponding structure of the pharmacophore Ph1 found relative to active compound **4**.

Compound **4** taken as template compound from active ones was accepted active one and from the template compound **4** an activity feature 1 (or pharmacophore, Ph1) were found. It’s given in Fig. (**2**), alone with the corresponding ETSCs, which describe electronic-topological characteristics of the fragments (see Fig. **2**).

In the matrices, the effective charges on atoms (local atomic characteristics, Q_i_) were chosen for diagonal elements, while at place of non-diagonal elements representing inter-atomic characteristics, there are either Wiberg’s indices [22], (W_ij_, for bonds) or optimized distances (R_ij_, in Å, for chemically non-bonded pairs of atoms). C_10_-C_12_ pairs of atoms are chemical bonded and bond order is 1.01 ē. The distance between C_12_ and N_15_ atom is 2.81 Å.

Sub-matrices given in Fig. (**2**) were found after setting some allowable limits for the comparison of matrix elements. For both series, the limits are δ_1_= 0.05 for diagonal elements of the ETMCs and δ_2_= 0.15 for their off-diagonal elements comparison. The pharmacophores found from the ETM-calculations are realized in *all *(15) active compounds. Statistical estimates for the pharmacophores are given in Table **1**.

An activity feature Ph1 is shown along with its ETSA of the order 8x8 (see Fig. **2**). Only its upper triangle is given because of the symmetry of bounds. The pharmacophore was found in 15 of 20 active compounds, n_A_, and it was found in 2 of 20 inactive compounds, n_IA_. Thus, the probability P_A_ of its realization in this class is about 0.84. As seen from the pharmacophore structure, the active feature 1, Ph1, consists of the 8 atoms (C_1_, C_5_, C_6_, C_9_, C_10_, C_12_, C_13_ and N_15_).

Compound **6** was chosen as template and compared the rest of the molecules in the series. Pharmacophores 2, Ph2, were found in 13 of 20 active compounds having the probability P_A_ = 0.88 as seen in Fig. (**3**).

Compound **19** was chosen as template and the active feature 3, Ph3, is formed by 4 different atoms and statistical estimates for the pharmacophores are given in see Fig. (**4**).

### Determination of Anti-Pharmacophores Features

**Fig. (5):**
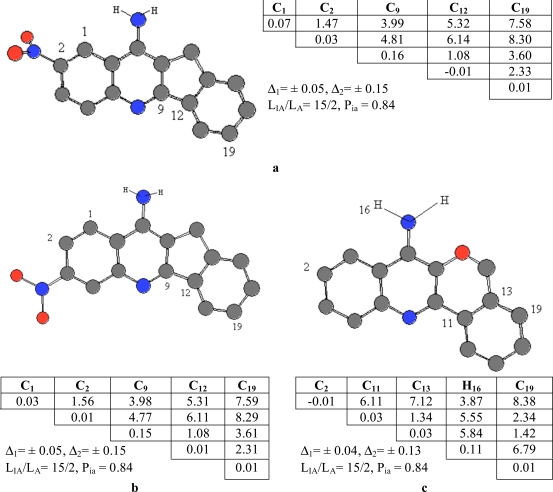
ETSC and corresponding structure of the pharmacophore: **(a)** APh1; **(b)** APh2; and **(c)** APh3 found relative to inactive molecule **25**, **27** and **42**

Anti-pharmacophores, alone with pharmacophores, are also of interest for the researches as those parts of molecules that are responsible for the considerable decrease or complete loss of the activity in view. To find anti-pharmaco-phores, inactive compounds **25**, **27** and **42** were selected as template compounds (their structures are given in Fig. **5**). Again, both protonated and unprotonated forms of compounds **25**, **27** and **42** were studied. As an example, APh1, APh2 and APh3 anti-pharmacophores are shown in the figure by their numbers, while corresponding sub-matrices are given nearby.

The anti-pharmacophore APh1, APh2 and APh3 (see Fig. **5**) consisting of 5 atoms, enters the structures of 15 inactive molecules and are found 2 active compounds (see Fig. **5**). The probability is 84%, because they are found in 15 from 20 inactive molecules in total.

When comparing the structures of the pharmacophores and anti-pharmacophores, one can pay attention to the differences in their spatial and electron characteristics. Thus, pharmacophores and anti-pharmacophores can play their role in the activity prediction only if both types of fragments participate in the process of prognosis. Thus, the set of activity/inactivity fragments found as the result of this study forms a basis for a system of the Human AChE activity binding affinity prediction.

As seen from Fig. (**6**), the pharmacophores Ph1-Ph3 and anti-pharmacophores APh1-APh3 found as the result of the *ETM *application were used as a basis for a system formation capable of the thiobenzamide and quinolizidine analogues activity prediction.

## CONCLUSION

A series of tacrine and 11 *H*-Indeno-[1,2-b]-quinolin-10-ylamine compounds demonstrating Human AChE activity binding affinity is studied by means of the ETM, which takes into account both structural and electronic characteristics of molecules. Based on pharmacophores and anti-pharmacophores calculated by the ETM-software as sub-matrices containing important spatial and quantum chemistry characteristics, a system for the activity prognostication is developed. The comparison of pharmacophores determined relative to the neutral and protonated forms was in favor of protonated forms as to their statistical estimates. The system was tested on a few compounds with molecular skeletons other than those that were characteristic of the training sets which allow to identify the presence/absence of the Human AChE activity binding affinity (with probabilities 84-89%) in molecules with diverse structures and predicting the level of the activity.

The initial data analysis reveals the intimate relation of the activity exhibition by compounds to their spatial and electronic states. Any changes in the values of the matrices that excel the limits allowed cause diminishing or complete loss of the activity.

The system for the Human AChE activity prediction is supposed to be used for new potent drugs synthesis which will make the screening of the new potential drugs’ design easy and effective.

## Figures and Tables

**Fig. (3):**
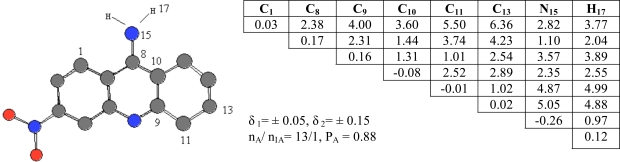
ETSC and corresponding structure of the pharmacophore Ph2 found relative to active compound **6**.

**Fig. (4):**
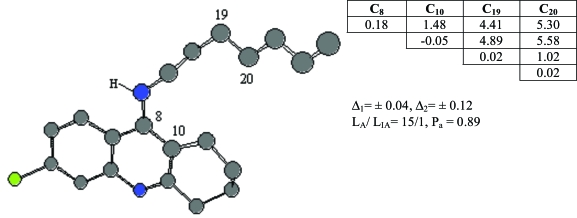
ETSC and corresponding structure of the pharmacophore Ph3 found relative to active compound **19**.

**Fig. (6):**
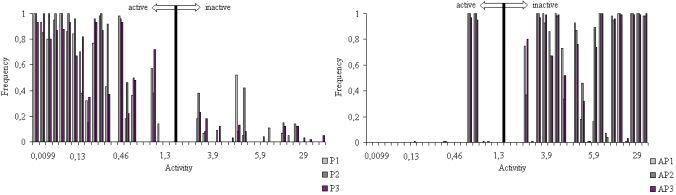
Frequency of the fragments’ occurrences in the compounds studied: for Pharmacophores Ph1, Ph2 and Ph3; for antipharmacophores APh1, APh2 and APh3.

**Table 1:** The Series of Chemical Compounds Under Investigation

**Skeleton I**								
Compound	R	R_1_	R_2_	R_3_	R_4_	R_5_	X	Activity, (IC_50_µ M)
**1**	H	Me	H	-	-	-	-	8.1
**2**	H	H	Me	-	-	-	-	0.10
**3**	H	Cl	H	-	-	-	-	0.55
**4**	H	H	Cl	-	-	-	-	0.0099
**5**	H	NO_2_	H	-	-	-	-	3.0
**6**	H	H	NO_2_	-	-	-	-	0.028
**7**	H	H	O-Me	-	-	-	-	0.35
**8**	H	NH_2_	H	-	-	-	-	3.8
**9**	H	H	F	-	-	-	-	0.087
**10**	H	Cl	Cl	-	-	-	-	0.47
**11**	H	O-Me	O-Me	-	-	-	-	5.2
**12**	CH_2_-Ph	Me	H	-	-	-	-	3.7
**13**	CH_2_-Ph	H	Me	-	-	-	-	0.75
**14**	CH_2_-Ph	H	Cl	-	-	-	-	0.17
**15**	CH_2_-Ph	NO_2_	H	-	-	-	-	1.6
**16**	CH_2_-Ph	H	NO_2_	-	-	-	-	4.8
**17**	C_7_H_15_	Me	H	-	-	-	-	0.39
**18**	C_7_H_15_	H	Me	-	-	-	-	0.13
**19**	C_7_H_15_	H	Cl	-	-	-	-	0.013
**20**	C_7_H_15_	H	NO_2_	-	-	-	-	0.29
**21**	C_7_H_15_	H	O-Me	-	-	-	-	0.46
**22**	C_7_H_15_	H	F	-	-	-	-	0.045
**23**	H	H	H	-	-	-	-	0.25
**Skeleton II**								
**Compound**	**R**	**R_1_**	**R_2_**	**R_3_**	**R_4_**	**R_5_**	**X**	**Activity, (IC_50_μ M)**
**24**	H	H	H	H	H	H	-	0.68
**25**	H	NO_2_	H	H	H	H	-	>100
**26**	H	NO_2_	H	H	H	H	-	5.9
**27**	H	H	NO_2_	H	H	H	-	67.5
**28**	H	H	NH_2_	H	H	H	-	29
**29**	H	H	Cl	H	H	H	-	6.5
**30**	H	H	F	H	H	H	-	1.2
**31**	H	H	H	O-Me	H	H	-	1.6
**32**	H	H	H	H	O-Me	H	-	6.5
**33**	H	H	H	H	H	O-Me	-	4.3
**34**	H	H	H	Me	H	H	-	3.9
**35**	H	H	H	H	H	Me	-	4.6
**36**	H	H	H	H	F	H	-	0.43
**37**	H	H	H	H	Cl	H	-	5.4
**38**	CH_2_-Ph	H	H	H	H	H	-	7.1
**39**	Me	H	H	H	H	H	-	1.3
**40**	C_7_H_15_	H	H	H	H	H	-	4.3
**Skeleton III**								
**Compound**	**R**	**R_1_**	**R_2_**	**R_3_**	**R_4_**	**R_5_**	**X**	**Activity, (IC_50_µ M)**
**41**	-	-	-	-	-	-	CH_2_-CH_2_	2.98
**42**	-	-	-	-	-	-	OCH_2_	245
**43**	-	-	-	-	-	-	SCH_2_	18.58
**44**	-	-	-	-	-	-	-	11.48
